# Searching for the needle in the haystack: deconvoluting the evolutionary dynamics of residual disease in human glioblastoma

**DOI:** 10.1093/annonc/mdz042

**Published:** 2019-02-07

**Authors:** S T C Shepherd, K Litchfield, S Turajlic

**Affiliations:** 1 The Renal and Melanoma Unit, The Royal Marsden NHS Foundation Trust, London; 2 Cancer Evolution and Genome Instability Laboratory, The Francis Crick Institute, London, UK

The evolution of divergent subpopulations of cancer cells within the same tumour has been proposed to underlie the development of treatment resistance and the recurrence of malignancy across multiple tumour types [1]. In this issue of *Annals of Oncology,* Spiteri et al. [2] utilise multi-region whole-exome sequencing to unravel the complex nature of cancer evolution in time and space that underlies glioblastoma (GBM) recurrence and offer novel insights into the phylogenetic relationships between the initial bulk tumour mass, clinically occult residual disease following initial radical therapy, and relapsed GBM.

GBM is the most common primary brain malignancy in adults characterised by a devastating prognosis and a lack of effective therapeutic options. Since the 1970s, treatment has consisted of maximal resection followed by focal external beam radiotherapy [3], and more recently concomitant temozolamide has seen modest improvements in outcomes, although even in selected clinical trial populations median survival remains just 14–15 months [3, 4].

Following initial radical therapy, tumour recurrence inevitably occurs and is the predominant source of mortality in these patients [3]. Clinical phenotypes of relapse vary; local relapse—within 2 cm of initial debulking surgery—occurs in the majority of cases, although up to a third of patients relapse with distal recurrence or with multifocal disease [5]. Indeed, diffuse parenchymal infiltration is a hallmark of GBM [6] and scattered tumour cells migrate throughout the substance of the brain along blood vessels [7] and white matter tracts [8] and are also present in the sub-ventricular zone (SVZ), a neural stem cell niche, at diagnosis [7, 8]. Recurrence is usually a substrate of this residual infiltrative disease and an understanding of the genomic events and evolutionary trajectories underlying these recurrence events are critical for improving patient care.

The genomic architecture of untreated GBM was revealed through genomic sequencing studies such as The Cancer Genome Atlas (TCGA) [9, 10] identifying distinct genetic and epigenetic alterations in several core oncogenic signalling pathways and distinct transcriptional profiles that allowed stratification of the disease into clinically relevant subtypes. However, these initial single-region profiling studies failed to capture to complexity of the genomic landscape in GBM and multi-region profiling of individual tumours revealed significant intratumoural heterogeneity at both the genomic and transcriptomic level [11].

Insights into the temporal evolution of GBM have been revealed by profiling matched therapy naive and recurrent tumours, revealing significant heterogeneity in both somatic mutations and copy number alterations at relapse [12]. Thus, salvage therapies targeting genomic and epigenomic changes seen at baseline can fail due to the expansion of minor subclones in the original tumour. It is crucially important, therefore, to identify and characterise these recurrence-initiating clones and their therapeutic vulnerabilities so that they can be targeted.

**Figure 1.**
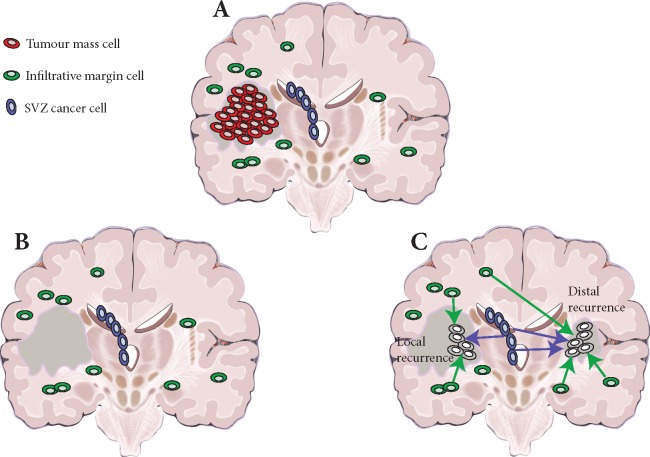
Residual disease in glioblastoma. (A) At surgery, only the primary tumour mass (red online) is removed (in dark grey the resection cavity). (B) However, infiltrative cells in the normal brain parenchyma (green online) and sub-ventricular zone (SVZ) (blue online) are left behind. (C) Residual glioblastoma cells infiltrated throughout the brain can give rise to relapse, both locally and distally. Reproduced this figure with permission from Spiteri et al. [2].

Spiteri et al. present their analysis [2] of 69 tissue samples collected from 10 patients with *IDH1* wildtype GBM and 1 patient with *IDH1* mutant anaplastic astrocytoma. They performed multi-region whole-exome sequencing from the primary tumour mass, SVZ and infiltrative margin collected using fluorescent guided resection and, in two cases, matched tissue from a second surgery at local relapse were available for comparison (Figure 1) [2].

In keeping with previous reports in this disease, they demonstrated intra-tumoural heterogeneity at the level of both somatic driver mutations and copy number alterations spatially within the bulk tumour mass at presentation and temporally at recurrence. They inferred the clonal relationship between the primary tumour mass and residual disease identified in the SVZ and the infiltrating margin and validated their observations of these relationships by utilising molecular clock haplotyping, which allows orthogonal reconstruction of the observed evolutionary relationships [13].

Their analyses suggest that tumour cells isolated from the residual disease in the infiltrative margin and the SVZ relate to early ancestral clones rather than the most advanced dominant clone in the primary tumour implying that the diffuse infiltration of cancer cells characteristic of GBM is an early event in tumorigenesis. In the two cases where tissue was available at relapse, the recurrent tumour bulk and GBM cells in the SVZ had acquired several new mutations, but no de novo mutations were detected at the infiltrative margin at recurrence.

The authors are to be commended for their presentation of this work which makes an important contribution to the growing body of evidence of evolutionary divergence and molecular diversity in this disease. Notably, Kim et al. [12] published an analysis of 38 primary/recurrence GBM pairs, observing that local recurrences typically retain a high proportion of genomic aberrations from the therapy naive tumour, whereas distal recurrences were characterised by branched patterns of evolution where the recurrent tumour undergoes a divergent evolutionary path from the most recent common ancestor. Intriguingly, Spiteri et al. observe these early branching patterns in the relationship between the primary tumour and the invasive margin implicating the clones present in the infiltrating margin as the substrate for distal relapse.

Importantly, the work from Spiteri et al. also corroborates the recent findings of Lee et al. [14] who published data derived from 28 patients with GBM, suggesting that the human SVZ harbours cells containing low-frequency GBM driver mutations that migrate to other parts of the brain and give rise to malignant glioma. These findings underlie the importance of fully characterising the residual disease in patients with GBM with the inherent ability to seed re-growth and resistance to rescue therapeutics.

Recent reports [15, 16] have indicated that personalised multi-epitope neoantigen vaccinations may be feasible for tumours such as glioblastoma, which typically have a relatively low mutational load and an immunologically ‘cold’ tumour microenvironment. The therapeutic success of such vaccination approaches will rely on the targeting of the recurrence-initiating clones that remain following initial radical therapy.

Spiteri et al. are to be congratulated for overcoming the various technical, ethical and logistical challenges associated with the conduct of longitudinal cohort studies in GBM. Given the difficulty of obtaining research material in these patients we agree that, as stated by the authors, future analyses should be complimented by post-mortem studies such as CASCADE (Cancer Tissue Collection after Death) and PEACE (Posthumous Evaluation of Advanced Cancer Environment, NCT03004755) which afford the highest resolution sampling to better understand the evolutionary history underlying this devastating disease and inform future therapeutic targets both molecular and immunogenic. 
